# ResT-IMU: A Two-Stage ResNet-Transformer Framework for Inertial Measurement Unit Localization

**DOI:** 10.3390/s25113441

**Published:** 2025-05-30

**Authors:** Yanping Zhu, Jianqiang Zhang, Wenlong Chen, Chenyang Zhu, Sen Yan, Qi Chen

**Affiliations:** 1 School of Wang Zheng Microelectronics, Changzhou University, Changzhou 213159, China; 13105506003@163.com (J.Z.) ; cczuchenwenlong@163.com (W.C.); 2 School of Computer Science and Artificial Intelligence, Changzhou University, Changzhou 213159, China; zcy@cczu.edu.cn (C.Z.); ys391317684@163.com (S.Y.); cczuchenqi@163.com (Q.C.)

**Keywords:** localization techniques, two-stage models, loss functions, attention mechanisms, trajectory prediction

## Abstract

To address the challenges of accurate indoor positioning in complex environments, this paper proposes a two-stage indoor positioning method, ResT-IMU, which integrates the ResNet and Transformer architectures. The method initially processes the IMU data using Kalman filtering, followed by the application of windowing to the data. Residual networks are then employed to extract motion features by learning the residual mapping of the input data, which enhances the model’s ability to capture motion changes and predict instantaneous velocity. Subsequently, the self-attention mechanism of the Transformer is utilized to capture the temporal features of the IMU data, thereby refining the estimation of movement direction in conjunction with the velocity predictions. Finally, a fully connected layer outputs the predicted velocity and direction, which are used to calculate the trajectory. During training, the RMSE loss is used to optimize velocity prediction, while the cosine similarity loss is employed for direction prediction. Theexperimental results demonstrate that ResT-IMU achieves velocity prediction errors of 0.0182 m/s on the iIMU-TD dataset and 0.014 m/s on the RoNIN dataset. Compared with the ResNet model, ResT-IMU achieves reductions of 0.19 m in ATE and 0.05 m in RTE on the RoNIN dataset. Compared with the IMUNet model, ResT-IMU achieves reductions of 0.61 m in ATE and 0.02 m in RTE on the iIMU-TD dataset and reductions of 0.32 m in ATE and 0.33 m in RTE on the RoNIN dataset. Compared with the ResMixer model, ResT-IMU achieves reductions of 0.13 m in ATE and 0.02 m in RTE on the RoNIN dataset. These improvements indicate that ResT-IMU offers superior accuracy and robustness in trajectory prediction.

## 1. Introduction

In recent years, localization technologies have become essential for a variety of applications, including navigation, tracking, and environmental perception. Accurate indoor localization is particularly crucial, as people spend a significant amount of time in indoor environments such as shopping centers, airports, and offices. Common indoor localization technologies, such as RFID [1], UWB [2], Wi-Fi positioning [3,4], Bluetooth positioning [5], infrared positioning [6], ultrasonic positioning [7], vision-based positioning [8], INS [9,10], geomagnetic positioning [11], and Zigbee positioning [12], each possess unique application scenarios and performance characteristics. However, many of these technologies necessitate additional hardware deployment. In contrast, Inertial Measurement Unit (IMU) technology, as an integrated component on modern smartphones, offers extensive application potential in GPS-limited scenarios by capturing high-precision motion states without the need for additional hardware. Depending on the implementation techniques, IMU localization can be categorized into traditional and deep learning-based approaches.

Traditional IMU localization typically employs methods such as step counting and zero-velocity updates to mitigate errors. Step counting involves monitoring gait information during walking or running to estimate position and distance by counting the number of steps taken. Zero-velocity updating identifies moments when the object is stationary and utilizes these instances to correct the velocity drift errors in the inertial navigation system (INS). Wang et al. [13] investigated the impact of IMU installation locations on the accuracy of pedestrian inertial navigation systems assisted by zero-velocity update techniques. Another approach involves using filters to combine IMU measurements with prior information to estimate position and velocity in real time, thereby correcting errors. The Kalman filter, a common example, effectively integrates the acceleration and angular velocity data from IMUs through its recursive data fusion algorithm. References [14,15,16,17] have investigated the use of filtering techniques in combination with IMU data to enhance the accuracy and robustness of navigation and positioning systems. Fusing IMU data with other sensor data is also a viable solution. For example, GPS provides globally accurate position information. When combined with IMU data, it can eliminate cumulative errors and reduce dependence on closed-loop systems. Magnetometers can measure the Earth’s magnetic field to assist the IMU in calibrating yaw angles, thereby reducing long-term accumulated errors and improving positioning accuracy. Eckenhoff et al. [18] proposed a multi-IMU, multi-camera visual-inertial navigation system named MIMC-VINS, which integrates information from all sensors.

Deep learning-based IMU localization leverages advanced machine learning techniques [19,20] to reduce errors in trajectory estimation from IMU data. Replacing traditional step prediction and zero-velocity prediction methods with neural networks is a viable approach. The advantage of this method lies in its ability to utilize deep learning models to learn and infer inertial odometry directly from raw IMU data, thereby simultaneously addressing errors in dynamic motion. This enhances the overall performance and reliability of the positioning system. Wagstaff et al. [21] and Brossard et al. [22] proposed methods to enhance the accuracy of zero-velocity aided inertial navigation systems by replacing the standard zero-velocity detector with a Long Short-Term Memory (LSTM) neural network. Neural networks can also be employed to dynamically adjust the parameters of filters to effectively mitigate the error issues associated with IMU localization. Brossard et al. [23] utilized deep neural networks to dynamically tune the noise parameters of filters, thereby enabling real-time determination of the most suitable covariance noise matrix.

Learning position transformations directly from IMU data is a method that warrants attention. Chen et al. [24,25] proposed a deep neural network framework that employs an LSTM model to learn position transformations from IMU data, thereby constructing an inertial odometry system. The LSTM model is capable of effectively modeling the temporal dynamics of human motion, thereby making it suitable for inertial navigation tasks. However, the sequential nature of LSTMs restricts their parallelization capabilities, leading to slower training speeds when dealing with very long sequences. Herath et al. [26] developed a ResNet-based deep learning architecture that focuses on extracting position information from IMU data to construct an inertial odometry system. However, ResNet itself may not fully capture the temporal dependencies crucial for inertial navigation, as it is primarily designed for extracting spatial features. Recently, the Transformer architecture [27] has emerged as a powerful alternative for sequence modeling. Unlike RNNs, the Transformer relies entirely on attention mechanisms to capture global dependencies between input and output. This allows for significantly more parallelization during training and can lead to superior performance in tasks such as machine translation. The Transformer’s ability to handle long-range dependencies and its efficiency in training make it a promising candidate for improving IMU localization [28].

This paper addresses the challenge of accurate indoor localization by proposing a novel two-stage network-based IMU localization model, named ResNet–Transformer Integrated Network for Inertial Measurement Units (ResT-IMUs). Unlike the two-stage methods in [29,30], which focus on switching between different localization techniques or fusing multiple sensor modalities, this method leverages deep learning techniques, combining ResNet and Transformer architectures to process IMU sensor data, learn human walking patterns, predict movement velocity, and calculate the trajectory of a target within an indoor environment. Specifically, the first stage of ResT-IMU predicts the instantaneous velocity from IMU data using a ResNet-based velocity branch model, while the second stage refines the estimation of the motion direction using a Transformer-based orientation branch model. The main contributions of this paper are as follows:

- This paper applies the Transformer architecture to motion trajectory prediction using IMU data. By leveraging the self-attention mechanism of the Transformer, it captures long-term dependencies in the IMU data. This not only improves the accuracy of the predictions but also enhances the model’s adaptability to complex dynamic environments.
- This paper proposes an innovative two-stage prediction framework. The model first predicts the instantaneous velocity and subsequently refines the estimation of the motion direction based on the predicted velocity. This staged approach enables the model to focus on specific prediction tasks at each stage, thereby enhancing the overall prediction accuracy and reliability.
- The model adaptively adjusts the size of the data window for direction prediction based on the predicted velocity. This dynamic adjustment mechanism enables the model to more flexibly handle different motion states, particularly during periods of drastic velocity changes, thereby allowing it to quickly focus on the most relevant data intervals. Additionally, velocity information is incorporated into the loss function for direction prediction, thereby making the loss calculation dependent not only on the accuracy of direction but also on the matching degree of velocity.

The structure of this paper is organized as follows: Section 2 introduces the proposed ResT-IMU model. Section 3 presents the experimental results. Finally, the paper concludes and discusses future work in Section 4.

## 2. Improved ResT-IMU Model

**Figure 1:**
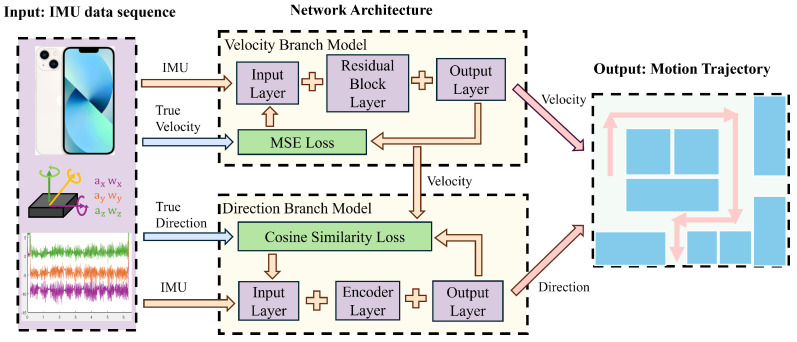
Architecture of the ResT-IMU model.

The architecture of the proposed ResT-IMU model is depicted in Figure 1. It takes the IMU acceleration and angular velocity, collected via a smartphone, as input. For the velocity branch, it employs a ResNet-based architecture to calculate velocity, thereby leveraging ResNet’s strong spatial feature extraction capabilities. For the direction branch, it uses a Transformer-based architecture to calculate direction, thereby taking advantage of the Transformer’s ability to handle long-range dependencies. This design integrates the strengths of existing techniques to address their limitations and enhance the accuracy of trajectory calculation.

The model architecture comprises two main components: the velocity branch model and the direction branch model. The core of the velocity branch model is the ResNet network. First, it maps the acceleration and angular velocity data from the IMU from the input space to the feature space, thereby generating high-dimensional feature vectors. Then, the output layer maps these high-dimensional feature vectors to the output space. The network parameters are optimized using the MSE loss function, resulting in the generation of the velocity sequence as the final output of this branch. The core of the direction branch model is the encoder structure within the Transformer network. The encoder receives input vectors that have been processed through an embedding layer and augmented with positional information. It captures the temporal sequence features of the data using the self-attention mechanism. The output layer then maps the high-dimensional feature vectors to the output space. In the cosine similarity loss function, the network parameters are optimized by incorporating the input data and the velocity information from the velocity branch model. This results in the generation of the direction sequence. By combining the velocity sequence and the direction sequence, the final trajectory of motion is computed.

### 2.1. Data Preparation

**Figure 2:**
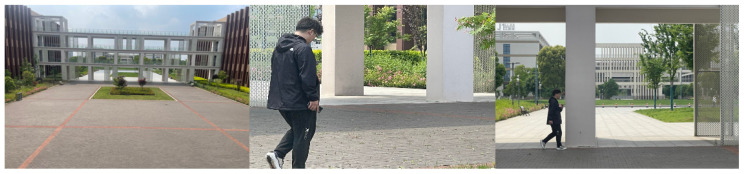
Experimental data collection setup.

The model in this paper was trained using the iIMU-TD dataset, which comprises a total of 2.41 h and 10.4 km of data, collected in both indoor and outdoor experimental environments. The dataset was captured using two smartphones. One smartphone, equipped with an IMU sensor, was used to capture the triaxial acceleration and triaxial angular velocity data during the experiments. The other smartphone used in the experiments was ASUS_A002A, which integrates Tango technology developed by Google. This technology provides localization tags for the trajectory through visual recognition, as shown in Figure 2. During the experiments, the data collector placed the Tango-enabled smartphone on the chest to record a more accurate trajectory. The other smartphone was held in hand in a manner that simulates everyday use, to collect IMU data at a sampling rate of 200 Hz. After the experiments, the raw IMU data were preprocessed through filtering and other operations and then saved for subsequent analysis and use.

In the experiments, the IMU data record dynamic information in the device coordinate system, rather than in the global coordinate system. To predict the trajectory of motion, these data must be transformed into the global coordinate system, as shown in the following Equations (1) and (2):

(1) $${\vec{a}}_{T_{i}}=R_{I_{i}}^{T_{i}}\cdot {\vec{a}}_{I_{i}}$$

(2) $${\vec{\omega }}_{T_{i}}=R_{I_{i}}^{T}\cdot {\vec{\omega }}_{I_{i}}$$

In the equations, ${\vec{a}}_{T_{i}}$ and ${\vec{\omega }}_{T_{i}}$ represent the acceleration and angular velocity vectors in the Tango device coordinate system at time *i*, while ${\vec{a}}_{I_{i}}$ and ${\vec{\omega }}_{I_{i}}$ represent the acceleration and angular velocity vectors in the IMU device coordinate system at time *i*. $R_{I_{i}}^{T_{i}}$ is the rotation matrix that transforms from the IMU device coordinate system to the Tango device coordinate system at time *i*. The quaternion representation of this rotation matrix is given by the following Equation (3):

(3) $$q_{I_{i}}^{T_{i}}=\left(q_{T_{0}}\cdot q_{I_{0}}^{T_{0}}\cdot q_{I_{0}}^{-1}\right)\cdot q_{I_{i}}$$

In the equation, $q_{I_{i}}^{T}$ represents the quaternion that transforms from the IMU device coordinate system to the Tango device coordinate system at time *i*. $q_{T_{0}}$ and $q_{I_{0}}$ respectively represent the initial attitudes of the IMU and Tango devices in their own coordinate systems. $q_{I_{0}}^{T_{0}}$ represents the initial rotation quaternion from the IMU device coordinate system to the Tango device coordinate system, which is determined by fixing the initial relative attitude of the two devices.

### 2.2. Velocity Branch Model

**Figure 3:**
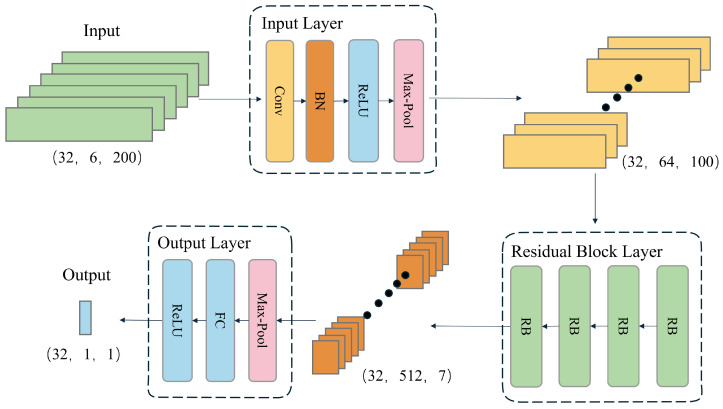
Architecture of the velocity branch model.

The velocity branch model is designed to predict the magnitude of instantaneous velocity from IMU data. Its architecture is depicted in Figure 3 and comprises three main components: the input module, the residual group module, and the output module. The input module first preprocesses the raw data collected by the IMU sensors. The data then flow through multiple residual blocks, which extract features by learning patterns in the data while mitigating the vanishing gradient problem using residual connections. Finally, the output module maps the extracted features to the predicted instantaneous velocity through global average pooling and a linear layer, thereby achieving accurate velocity estimation.

The velocity branch model employs a time window of 200 data points as input, based on the data collected by the IMU sensors, to predict the instantaneous velocity of the last frame in the sequence. To adapt to the characteristics of IMU data, which are one-dimensional sequences, the model utilizes one-dimensional convolutional layers and pooling layers. These layers are specifically designed to process one-dimensional data efficiently. Additionally, a ReLU activation function is added to the output layer to ensure that the predicted velocity values are non-negative, which is essential for regression tasks involving speed magnitude. These modifications not only enhance the model’s suitability for handling long sequences of IMU data but also reduce the parameter count, making the model more efficient and better suited for processing extensive data sequences. With a stride of 10 for step-by-step prediction, the model can transform an IMU data sequence of length *N* into a velocity magnitude sequence of length (N−200)/10. By applying this model, a series of discrete velocity values can be estimated from continuous IMU measurements, where each velocity value represents the speed magnitude of the last frame in a window of 200 data points.

#### 2.2.1. Input Layer

The input layer performs preliminary processing on the input data and extracts initial features. A one-dimensional convolutional layer with a kernel size of 7 increases the number of input data channels from 6 to 64. Subsequently, a batch normalization layer normalizes the output of the convolutional layer, which accelerates training and enhances model stability. The normalized data are then activated by the ReLU activation function to introduce non-linearity. Finally, a max-pooling layer reduces the temporal dimension of the features by half while preserving important features.

#### 2.2.2. Residual Block Layer

The model comprises four residual blocks. The number of output channels in each residual block increases according to the residual rules. After passing through the four residual blocks, the number of channels increases from 64 to 512. The addition of convolutional layers with a stride of 2 in the residual blocks reduces the temporal dimension from 100 to 7. This enables the model to extract more advanced features while removing redundant information, enhancing the model’s generalization ability.

#### 2.2.3. Output Layer

The output layer maps the output features from the residual group to the final output space. The global average pooling layer compresses the temporal dimension of the features to 1, yielding the global average value for each channel. Subsequently, the fully connected layer regresses the 512-dimensional features to produce the final velocity prediction.

#### 2.2.4. Loss Function

To objectively evaluate the model’s performance in the velocity prediction task, the MSE is adopted as the primary metric for quantifying the error. MSE is a widely used measure for comparing the differences between model predictions and actual observations, especially in continuous numerical prediction tasks such as velocity estimation. The formula for calculating the velocity loss using MSE is shown in the following Equation (4):

(4) $$L\left(\nu \right)=\frac{1}{n}\sum_{i=1}^{n}{\left({\hat{\nu }}_{i}-\nu_{i}\right)}^{2}$$

In the equation, *n* denotes the number of samples, ${\hat{\nu }}_{i}$ represents the predicted velocity value for the i-th sample by the model, and $\nu_{i}$ is the corresponding actual velocity value. The smaller the value of MSE, the higher the prediction accuracy of the model, indicating a smaller difference between the predicted values and the actual values.

### 2.3. Direction Branch Model

**Figure 4:**
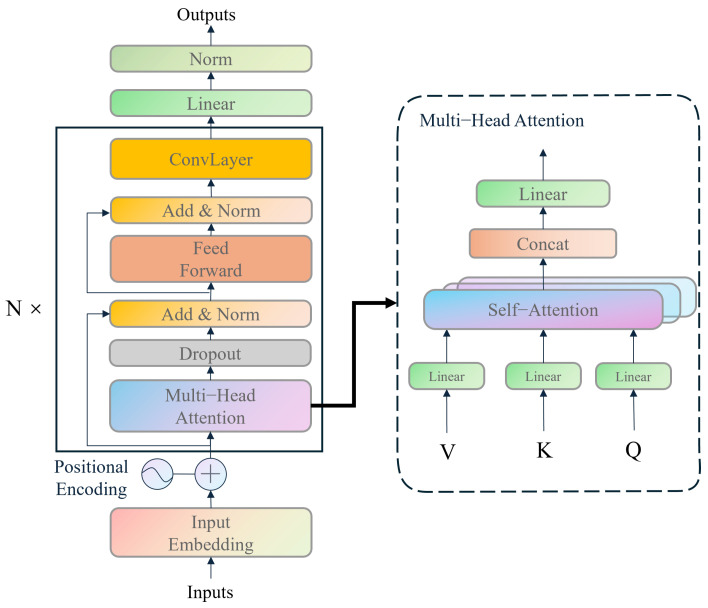
Architecture of the direction branch model.

The direction branch model is designed to predict the instantaneous direction vector from IMU data, taking into account the velocity magnitude predicted by the velocity branch model. The velocity magnitude is used not only for the dynamic adjustment of the data window but also in the loss function calculation. The model’s architecture, depicted in Figure 4, includes an input embedding layer, a positional encoding layer, an encoder layer, a fully connected layer, and an activation function. The embedding layer transforms the input into a higher-dimensional vector form, while the positional encoding layer adds a positional vector to each position. The encoder layer processes the input data through a multi-layer structure, with each head in the multi-head attention mechanism module performing self-attention computations, enabling the model to focus on the internal information of the sequence data. The fully connected layer converts the encoder’s output into a predicted direction vector, and the activation function introduces non-linearity to enhance the model’s expressive power. The proposed framework incorporates several novel modifications. Specifically, the decoder component of the Transformer architecture is omitted, as the objective is to derive an average velocity rather than predict a sequence of velocities within a window. This modification significantly enhances computational efficiency. Building on the insights from [31], a ConvLayer is integrated after each encoder layer to mitigate computational overhead and augment the model’s capacity to handle long sequence data. Ultimately, a linear layer and L2 normalization are appended after the encoder to generate the unit vector of velocity.

The direction branch model also employs a time window of 200 data points as input, based on the data collected by the IMU sensors, to predict the direction vector of the last frame in the sequence. Unlike the velocity branch model, it incorporates the velocity magnitude sequence to aid in direction prediction. Specifically, it discards certain data points based on the velocity magnitude, thereby dynamically adjusting the size of the window. With a stride of 10 for step-by-step prediction, the model can transform an IMU data sequence of length *N* into a direction vector sequence of length (N−200)/10. Finally, by combining the velocity magnitude sequence, a complete velocity vector sequence is formed. By applying this model, a series of discrete velocity vectors can be estimated from continuous IMU measurements, where each velocity vector represents the velocity of the last frame in a window of 200 data points.

#### 2.3.1. Embedding Layer

The embedding layer employs a convolutional layer with a kernel size of 3 to increase the number of input channels from 6 to 64, capturing local features within the time series and thereby enhancing the model’s expressive capability.

#### 2.3.2. Positional Encoding

The role of positional encoding is to provide the model with positional information for each time step, thereby enabling the model to capture the sequential order of the series. The calculation formula is shown as follows:

(5) $$PE\left(pos,2i\right)=\sin\left(\frac{pos}{10000^{2i/d_{\mathrm{model}}}}\right)\;\mathrm{and}\;PE\left(pos,2i+1\right)=\cos\left(\frac{pos}{10000^{2i/d_{\mathrm{model}}}}\right)$$

where pos is the position in the sequence, *i* is the dimension, and $d_{\mathrm{model}}$ is the dimensionality of the embedding.

#### 2.3.3. Encoder Layer

The encoder consists of multiple encoder layers, each of which contains an attention layer that performs self-attention operations on the input sequence to extract the dependencies within the time series. The multi-head attention mechanism module in each encoder layer has multiple heads, each of which executes the self-attention mechanism to focus on different parts of the sequence data. The calculation is shown in the following Equation (6):

(6) $$\mathrm{Attention}\left(Q,K,V\right)=\mathrm{softmax}\left(\frac{QK^{T}}{\sqrt{d_{k}}}\right)V$$

where *Q*, *K*, and *V* are the query, key, and value matrices, respectively, and $d_{k}$ is the dimension of the key vectors. The fully connected layer transforms the output of the encoder into the predicted direction vector, and the activation function introduces non-linearity, thereby enhancing the model’s expressive capability.

#### 2.3.4. Adaptive Window Adjustment

**Figure 5:**
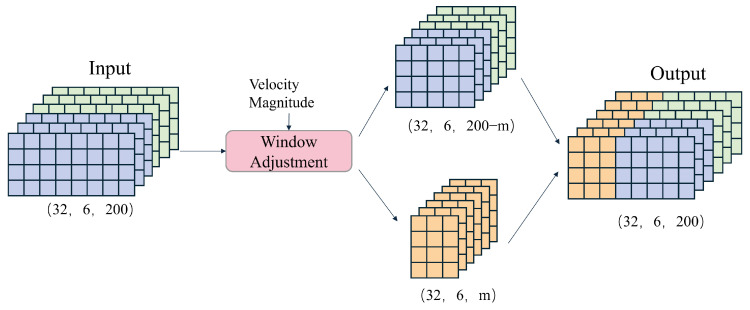
Dynamic window adjustment diagram.

This paper proposes a dynamic window adjustment strategy, illustrated in Figure 5, to enhance the accuracy of velocity prediction based on IMU data. This strategy dynamically adjusts the size of the data window based on the predicted velocity magnitudes, thereby optimizing computational efficiency and prediction accuracy.

The core idea of this strategy is to utilize the velocity prediction results from the preceding branch model to adjust the size of the data window in the direction branch model, thereby better capturing the motion features and enhancing prediction accuracy. The predicted velocities from the velocity branch model are first stored locally. Then, in the direction branch model, the size of the data window used for velocity direction prediction is dynamically adjusted based on the magnitude of velocity. This process is implemented through the following Equation (7):

(7) $$y_{j}=\left\{\begin{array}{cc} 0 & if\;0\leq j\leq m\\ x_{j} & if\;m\leq j\leq 200 \end{array}\right.$$

In the equation, $x_{j}$ and $y_{j}$ represent the data of the j-th frame within a 200-frame window, m denotes the adjustment node, and its calculation is shown in the following Equation (8):

(8) $$m=\left\{\begin{array}{cc} 100\times \nu & if\;\nu \leq 1\\ 100 & if\;\nu >1 \end{array}\right.$$

In the equation, *v* represents the velocity magnitude corresponding to the window.

The adjustment method takes the predicted velocity magnitude and the window data for direction prediction as inputs and outputs of the modified window data. Essentially, it does not change the window size but sets the values of the first *m* data points in the window to zero. The value of *m* increases with the velocity magnitude. This is because higher velocities are more likely to result in velocity changes, reducing the correlation between the current velocity and previous data points.

#### 2.3.5. Loss Function

For the direction branch model, this paper proposes a composite loss function that integrates cosine similarity and the magnitude of the velocity vector to optimize the network’s prediction accuracy of the velocity vector direction, especially at higher speeds. The construction of this loss function is based on the following theoretical foundation: during high-speed motion, deviations in direction prediction can significantly impact the accuracy of the trajectory. Therefore, the objective of this function is to enable the network to focus more on reducing directional prediction errors for samples with higher velocity values during the training process, as shown in the following Equation (9):

(9) L(θ)=(1−cos(θ))·|ν|

By multiplying the cosine similarity with the velocity magnitude, the model ensures that both the direction and magnitude of the velocity are considered during training. Let x be the predicted velocity direction vector and y be the true velocity direction vector. The cosine similarity is calculated as shown in the following Equation (10):

(10) $$\cos\left(\theta \right)=\frac{V_{pred}\cdot V_{true}}{∥V_{pred}∥\cdot ∥V_{true}∥}$$

## 3. Results

### 3.1. Velocity Prediction Error

The velocity branch model is capable of predicting the magnitude of velocity at each moment. This prediction serves not only to determine the mode of motion but also as a crucial metric for evaluating model performance, providing guidance for model assessment and optimization. The RoNIN dataset [26], created to support data-driven inertial navigation research, contains over 42.7 h of IMU sensor data and corresponding ground-truth 3D trajectory data from more than 100 different human subjects. These data were collected in various buildings, covering 276 sequences. In this section, experiments are conducted on different models using both the RoNIN dataset and the iIMU-TD dataset. Table 1 presents a comparison of different models in velocity prediction. The ResNet and LSTM network models are reproduced based on the work in [26], while the idea of the Encoder model is inspired by [28]. Additionally, the IMUNet [32] and ResMixer [33] models, both of which are based on the work in [26], represent the latest research in this area.

As shown in Table 1, the velocity branch of the ResT-IMU model outperforms single-stage models in predicting velocity magnitude. Single-stage models, which consider both the magnitude and direction of velocity during training, may yield less accurate predictions of velocity magnitude compared with models that focus solely on predicting velocity magnitude, such as the velocity branch of the ResT-IMU model. On the iIMU-TD and RoNIN datasets, the ResT-IMU model achieves average prediction errors of 0.0182 m/s and 0.014 m/s, respectively, corresponding to percentage errors of approximately 1.55% and 1.91%. Compared with other models, ResT-IMU demonstrates significantly lower error rates. On the iIMU-TD dataset, ResT-IMU outperforms ResNet (0.0265 m/s, 2.26%), LSTM (0.0881 m/s, 7.51%), Encoder (0.0281 m/s, 2.39%), IMUNet (0.0293 m/s, 2.51%), and ResMixer (0.0246 m/s, 2.11%). On the RoNIN dataset, ResT-IMU outperforms ResNet (0.0225 m/s, 3.08%), LSTM (0.0294 m/s, 4.02%), Encoder (0.0241 m/s, 3.29%), IMUNet (0.0233 m/s, 3.11%), and ResMixer (0.0221 m/s, 2.92%). These results highlight the superior performance of ResT-IMU in velocity prediction, achieving the lowest error rates among all tested models.

### 3.2. Absolute Error and Relative Error

After obtaining the velocity sequence from the velocity branch model and inputting it along with the original IMU data into the direction branch model, the direction sequence can be obtained through training. By combining the velocity sequence and the direction sequence into a velocity vector sequence, the trajectory of the target can be derived. Comparisons were made on the RoNIN dataset and the iIMU-TD dataset for the ResNet, LSTM, Encoder, IMUNet, and ResMixer models that predict velocity vectors, as well as the proposed ResT-IMU model. Relative trajectory error and absolute trajectory error were used as evaluation metrics. The RTE calculates the positioning error for each trajectory point over the subsequent 1 minute, sums these errors, and then divides the result by the total number of trajectory points. The ATE calculates the average error value for all trajectory points. By considering both error metrics, a comprehensive evaluation and comparison of the localization accuracy of various models can be made. Table 2 presents a comparison of trajectory errors for the six models on the two different datasets.

As shown in Table 2, the ResT-IMU model demonstrates superior performance in terms of absolute and relative trajectory errors on both the iIMU-TD and RoNIN datasets. Specifically, on the iIMU-TD dataset, the ResT-IMU model achieves the lowest ATE of 3.08 and the third-lowest RTE of 3.89, outperforming the ResNet, LSTM, Encoder, IMUNet, and ResMixer models. On the RoNIN dataset, the ResT-IMU model exhibits the lowest ATE of 3.64 and the lowest RTE of 2.60. The ResT-IMU model achieves the lowest error on both the iIMU-TD and RoNIN datasets, attributable to its two-stage analysis approach. This approach first predicts the velocity magnitude using a dedicated velocity branch, which focuses solely on the magnitude of the velocity. This dedicated branch allows the model to more accurately estimate the speed of the target, reducing the error in the velocity predictions. The direction branch then uses the predicted velocity magnitude along with the original IMU data to predict the direction of movement. By separating the prediction of velocity magnitude and direction, the model can more effectively capture the spatial and temporal features of the IMU data, leading to improved localization accuracy. For instance, compared with the ResNet model, the ResT-IMU model achieves a 5.21% reduction in ATE and a 4.41% reduction in RTE on the RoNIN dataset. On the iIMU-TD dataset, the ResT-IMU model achieves a 16.53% reduction in ATE and a 0.51% reduction in RTE compared with the IMUNet model. On the RoNIN dataset, the ResT-IMU model outperforms the IMUNet model with an 8.08% reduction in ATE and an 11.26% reduction in RTE. Additionally, compared with the ResMixer model, the ResT-IMU model achieves a 3.20% reduction in ATE and a 1.14% reduction in RTE on the RoNIN dataset.

**Figure 6:**
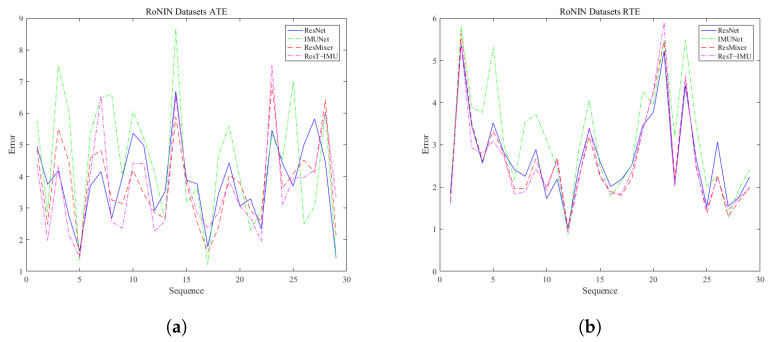
Error distribution chart of four models on the RoNIN dataset. (**a**) shows the ATE of each trajectory for four different models on the RoNIN dataset, while (**b**) shows the RTE of each trajectory for four different models on the RoNIN dataset, where the pink line corresponds to the proposed ResT-IMU model.

Figure 6 illustrates the error distribution of four network models on the RoNIN dataset. The pink line denotes the novel ResT-IMU model proposed in this study, while the other lines represent the ResNet, IMUNet, and ResMixer models. In terms of ATE, the ResT-IMU model outperforms the ResNet model in 20 of 29 trajectories, accounting for 69.0% of the total trajectories. It also outperforms the IMUNet model in 19 of 29 trajectories, equivalent to 65.5%. Furthermore, it surpasses the ResMixer model in 18 of 29 trajectories, representing 62.1% of the total trajectories. In terms of RTE, the ResT-IMU model outperforms the ResNet model in 21 of 29 trajectories, accounting for 72.4% of the total trajectories. It also outperforms the IMUNet model in 23 of 29 trajectories, equivalent to 79.3%. Additionally, it outperforms the ResMixer model in 17 of 29 trajectories, which is 58.6% of the total trajectories.

**Figure 7:**
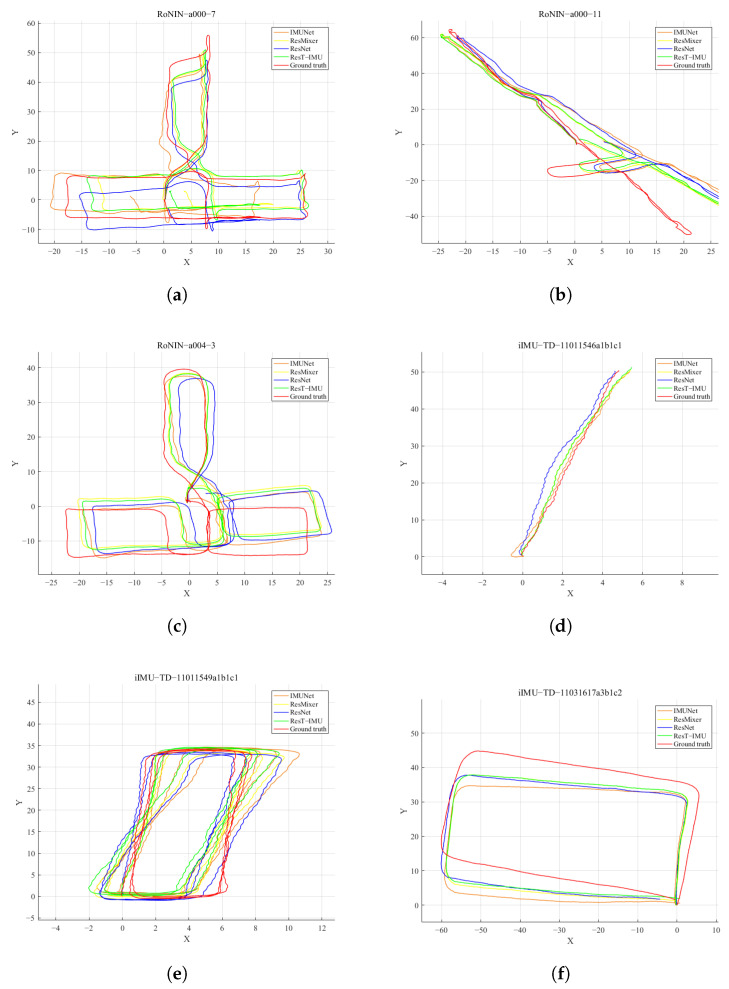
Trajectory comparison chart of different models on two datasets. (**a**–**c**) show the comparison of predicted trajectories and ground-truth trajectories for three data samples from the RoNIN dataset across different models. Similarly, (**d**–**f**) show the comparison of predicted trajectories and ground-truth trajectories for three data samples from the iIMU-TD dataset across different models. The red lines represent the ground-truth trajectories; the green lines correspond to the proposed ResT-IMU model.

Figure 7 depicts the trajectories of four models on partial data from the RoNIN dataset and the iIMU-TD dataset. In the figure, the red trajectory represents the ground truth, while the green trajectory corresponds to the novel model proposed in this study, which is compared with the trajectories of other models represented by different colors. Due to the presence of errors, noise, and bias in the IMU, the double integration algorithm accumulates significant errors over time, leading to substantial deviations from the true trajectory even in a short period. Neural network-based methods effectively reduce the accumulation of errors and provide accurate estimations of the motion trajectories. As shown in Figure 7a, the ATEs of the ResNet, IMUNet, ResMixer, and ResT-IMU models on the trajectory prediction are 2.92 m, 4.15 m, 2.86 m, and 2.26 m, respectively. When the motion reaches the upper region of the trajectory, the trajectory predicted by the ResT-IMU model is significantly closer to the ground truth. As shown in Figure 7f, the ATEs of the four models are 4.07 m, 4.45 m, 4.06 m, and 3.90 m, respectively. The endpoint of the trajectory predicted by the ResT-IMU model is closer to the endpoint of the ground truth. The velocity branch of the model ensures that the overall trajectory scale is closer to reality, while the direction branch enhances the model’s fitting accuracy during both straight-line motion and turns. These features indicate that the new model achieves higher accuracy and stronger robustness in trajectory prediction.

### 3.3. Cross-Device Adaptability of the Model

To verify the generalization ability of the network model designed for smartphone IMU data in other application scenarios, additional experiments were conducted on the TLIO dataset [34]. This dataset was collected from a head-mounted device equipped with a Bosch BMI055 IMU sensor, capturing diverse motion patterns through a series of real-world activities. The dataset comprises over 400 sequences, totaling approximately 60 h of pedestrian data, covering a wide range of activities, including walking, standing, doing household chores, playing billiards, and climbing stairs. Table 3 presents a comparison of different models in terms of trajectory error on the this dataset.

As shown in Table 3, the ResT-IMU model achieves nearly 2 m errors in both absolute and relative terms on the TLIO dataset. The absolute error is second only to the RoNIN (ResNet) algorithm, while the relative error outperforms other neural network methods. These results demonstrate that the proposed ResT-IMU model maintains high accuracy in other application scenarios, such as head-mounted IMU devices.

## 4. Conclusions

This paper presents a novel two-stage motion trajectory prediction model, ResT-IMU, which effectively enhances the accuracy and robustness of IMU-based localization. The proposed method leverages the self-attention mechanism of the Transformer architecture to capture temporal sequence features in IMU data, first predicting instantaneous velocity and then refining the estimation of motion direction based on the predicted velocity. The ResT-IMU model introduces a two-stage prediction framework that separates the prediction of instantaneous velocity and motion direction, allowing the model to focus on specific prediction tasks at each stage and thereby improving overall prediction accuracy and reliability. By combining ResNet for velocity prediction and Transformer for direction prediction, the model captures both spatial and temporal dependencies in IMU data, significantly improving trajectory prediction accuracy. The model also dynamically adjusts the size of the data window for direction prediction based on the predicted velocity, enabling it to handle different motion states more flexibly, especially during periods of drastic velocity changes.

Experimental results on the RoNIN and iIMU-TD datasets demonstrate that ResT-IMU outperforms existing models in both ATE and RTE. On the iIMU-TD dataset, ResT-IMU achieves an ATE of 3.08 and an RTE of 3.89, outperforming ResNet (ATE: 3.27, RTE: 3.73), LSTM (ATE: 3.87, RTE: 4.37), Encoder (ATE: 3.77, RTE: 3.95), IMUNet (ATE: 3.69, RTE: 3.91), and ResMixer (ATE: 3.21, RTE: 3.71). On the RoNIN dataset, ResT-IMU achieves an ATE of 3.64 and an RTE of 2.60, outperforming ResNet (ATE: 3.84, RTE: 2.72), LSTM (ATE: 4.22, RTE: 2.73), Encoder (ATE: 3.80, RTE: 2.78), IMUNet (ATE: 3.96, RTE: 2.93), and ResMixer (ATE: 3.76, RTE: 2.63). These results highlight the superior performance of ResT-IMU in reducing both ATE and RTE, thereby improving the overall accuracy of trajectory prediction.

Additional experiments on the TLIO dataset verify the model’s generalization ability in other application scenarios, with ResT-IMU achieving nearly 2 m errors in both absolute and relative terms, outperforming other neural network methods. Future research will focus on further optimizing the IMU-based localization algorithm, exploring more efficient network structures and training strategies to improve localization accuracy and model generalization ability, and reducing the algorithm’s power consumption to extend the device’s battery life while maintaining localization accuracy.

## Figures and Tables

**Table 1:** Comparison of velocity prediction errors for six models on two datasets.

	ResNet	LSTM	Encoder	IMUNet	ResMixer	ResT-IMU
iIMU-TD	0.0265	0.0881	0.0281	0.0293	0.0246	0.0182
RoNIN	0.0225	0.0294	0.0241	0.0233	0.0221	0.014

**Table 2:** Comparison of absolute and relative trajectory errors for six models on two datasets.

		ResNet	LSTM	Encoder	IMUNet	ResMixer	ResT-IMU
iIMU-TD	ATE	3.27	3.87	3.77	3.69	3.21	3.08
	RTE	3.73	4.37	3.95	3.91	3.71	3.89
RoNIN	ATE	3.84	4.22	3.80	3.96	3.76	3.64
	RTE	2.72	2.73	2.78	2.93	2.63	2.60

**Table 3:** Comparison of absolute and relative trajectory errors for six models on the TLIO datasets.

	ResNet	LSTM	Encoder	IMUNet	ResMixer	ResT-IMU
ATE	2.09	8.31	2.47	2.37	2.17	2.13
RTE	2.18	6.21	2.57	2.49	2.21	2.07

## Data Availability

The data presented in this study are available on request from the corresponding author. The data are not publicly available due to considerations of privacy protection and ethical principles.

## References

[1] Yao C.Y., Hsia W.C. (2018). An Indoor Positioning System Based on the Dual-Channel Passive RFID Technology. IEEE Sens. J..

[2] Bregar K. (2023). Indoor UWB Positioning and Position Tracking Data Set. Sci. Data.

[3] Shang S., Wang L. (2022). Overview of WiFi fingerprinting-based indoor positioning. IET Commun..

[4] Ma Z., Shi K. (2023). Few-Shot learning for WiFi fingerprinting indoor positioning. Sensors.

[5] De B.G., Quesada-Arencibia A., Garcia C.R., Rodriguez-Rodriguez J.C., Moreno-Diaz R. (2018). A Protocol-Channel-based Indoor Positioning Performance Study for Bluetooth Low Energy. IEEE Access.

[6] Tsai C.Y., Lin Y.W., Ku H.M., Lee C.Y. (2023). Positioning System of Infrared Sensors Based on ZnO Thin Film. Sensors.

[7] Khyam M.O., Xinde L., Ge S.S., Pickering M.R. (2017). Multiple Access Chirp-Based Ultrasonic Positioning. IEEE Trans. Instrum. Meas..

[8] Qin J., Li M., Li D., Zhong J., Yang K. (2022). A survey on visual navigation and positioning for autonomous UUVs. Remote Sens..

[9] Lu C., Uchiyama H., Thomas D., Shimada A., Taniguchi R.I. (2019). Indoor Positioning System Based on Chest-Mounted IMU. Sensors.

[10] Sun M., Wang Y., Joseph W., Plets D. (2022). Indoor localization using mind evolutionary algorithm-based geomagnetic positioning and smartphone IMU sensors. IEEE Sens. J..

[11] Kuo Y.H., Wu E.H.K. (2023). Intelligent geomagnetic indoor positioning system. Electronics.

[12] Uradzinski M., Guo H., Liu X., Yu M. (2017). Advanced indoor positioning using zigbee wireless technology. Wirel. Pers. Commun..

[13] Wang Y., Askari S., Shkel A.M. (2019). Study on mounting position of IMU for better accuracy of ZUPT-aided pedestrian inertial navigation. Proceedings of the 2019 IEEE International Symposium on Inertial Sensors and Systems (INERTIAL).

[14] Yang C., Shi W., Chen W. (2019). Robust M–M unscented Kalman filtering for GPS/IMU navigation. J. Geod..

[15] Chen J., Zhou B., Bao S., Liu X., Gu Z., Li L., Zhao Y., Zhu J., Li Q. (2021). A data-driven inertial navigation/Bluetooth fusion algorithm for indoor localization. IEEE Sens. J..

[16] Feng D., Wang C., He C., Zhuang Y., Xia X.G. (2020). Kalman-filter-based integration of IMU and UWB for high-accuracy indoor positioning and navigation. IEEE Internet Things J..

[17] Xu X., Xu X., Zhang T., Wang Z. (2018). In-motion filter-QUEST alignment for strapdown inertial navigation systems. IEEE Trans. Instrum. Meas..

[18] Eckenhoff K., Geneva P., Huang G. (2021). MIMC-VINS: A versatile and resilient multi-IMU multi-camera visual-inertial navigation system. IEEE Trans. Robot..

[19] Zhu C., Wang Q., Xie Y., Xu S. (2024). Multiview latent space learning with progressively fine-tuned deep features for unsupervised domain adaptation. Inf. Sci..

[20] Zhu C., Zhang L., Luo W., Jiang G., Wang Q. (2025). Tensorial multiview low-rank high-order graph learning for context-enhanced domain adaptation. Neural Netw..

[21] Wagstaff B., Kelly J. (2018). LSTM-based zero-velocity detection for robust inertial navigation. Proceedings of the 2018 International Conference on Indoor Positioning and Indoor Navigation (IPIN).

[22] Brossard M., Barrau A., Bonnabel S. (2019). RINS-W: Robust inertial navigation system on wheels. Proceedings of the 2019 IEEE/RSJ International Conference on Intelligent Robots and Systems (IROS).

[23] Brossard M., Barrau A., Bonnabel S. (2020). AI-IMU dead-reckoning. IEEE Trans. Intell. Veh..

[24] Chen C., Lu X., Markham A., Trigoni N. Ionet: Learning to cure the curse of drift in inertial odometry. Proceedings of the AAAI Conference on Artificial Intelligence.

[25] Chen C., Zhao P., Lu C.X., Wang W., Markham A., Trigoni N. (2020). Deep-learning-based pedestrian inertial navigation: Methods, data set, and on-device inference. IEEE Internet Things J..

[26] Herath S., Yan H., Furukawa Y. (2020). Ronin: Robust neural inertial navigation in the wild: Benchmark, evaluations, & new methods. Proceedings of the 2020 IEEE International Conference on Robotics and Automation (ICRA).

[27] Vaswani A., Shazeer N., Parmar N., Uszkoreit J., Jones L., Gomez A.N., Kaiser Ł., Polosukhin I. (2017). Attention is all you need. Adv. Neural Inf. Process. Syst..

[28] Herath S., Caruso D., Liu C., Chen Y., Furukawa Y. Neural inertial localization. Proceedings of the IEEE/CVF Conference on Computer Vision and Pattern Recognition.

[29] Yilmaz A., Temeltas H. (2024). A multi-stage localization framework for accurate and precise docking of autonomous mobile robots (AMRs). Robotica.

[30] Zhang L., Bao J., Xu Y., Wang Q., Xu J., Li D. (2022). From coarse to fine: Two-stage indoor localization with multisensor fusion. Tsinghua Sci. Technol..

[31] Zhou H., Zhang S., Peng J., Zhang S., Li J., Xiong H., Zhang W. Informer: Beyond efficient transformer for long sequence time-series forecasting. Proceedings of the AAAI Conference on Artificial Intelligence.

[32] Zeinali B., Zanddizari H., Chang M.J. (2024). Imunet: Efficient regression architecture for inertial imu navigation and positioning. IEEE Trans. Instrum. Meas..

[33] Lai R., Tian Y., Tian J., Wang J., Li N., Jiang Y. (2024). ResMixer: A lightweight residual mixer deep inertial odometry for indoor positioning. IEEE Sens. J..

[34] Liu W., Caruso D., Ilg E., Dong J., Mourikis A.I., Daniilidis K., Kumar V., Engel J. (2020). Tlio: Tight learned inertial odometry. IEEE Robot. Autom. Lett..

